# What have studies using finite element analysis taught us about the diabetic foot? A systematic review

**DOI:** 10.1186/1757-1146-7-S1-A82

**Published:** 2014-04-08

**Authors:** Scott Telfer, Ahmet Erdemir, James Woodburn, Peter R Cavanagh

**Affiliations:** 1 Institute for Applied Health Research, Glasgow Caledonian University, Glasgow, UK; 2 Department of Orthopaedics and Sports Medicine, University of Washington, Seattle, WA, USA; 3 Computational Biomodeling (CoBi) Core and Department of Biomedical Engineering, Cleveland Clinic, Cleveland, OH, USA

## Background

Over the past two decades finite element (FE) analysis has become a popular tool for researchers looking to simulate the biomechanics of the foot in people with diabetes. The ultimate aims of these simulations have been to improve understanding of the complicated nature of mechanical loading and to inform interventions designed to prevent plantar ulceration. This review is intended to provide a systematic review of these FE analysis based computational simulations.

## Methods

PUBMED, ScienceDirect and Web of Science databases were searched for relevant peer reviewed articles using the keywords “diabetic foot”, “finite element” and related synonyms. This review considered original research studies that utilised FE models of the foot or part of the foot to simulate function, tissue behaviour, or structural deformities associated with the diabetic foot. In addition, studies using FE to study footwear, insoles or surgical interventions intended to reduce ulceration risk in the diabetic foot were eligible for inclusion.

## Results

Thirty relevant articles were found covering three primary themes: investigations of the characteristics of the diabetic foot (10 articles); design of interventions to reduce ulceration risk (17 articles); and methodological aspects for modeling the diabetic foot (3 articles). Several FE studies have provided estimates of external and internal soft tissue loading, and suggested that internal stresses may often be considerably larger than those measured at the plantar surface and are proportionally greater in the diabetic foot compared to controls. A series of results that inform the design of insoles, footwear and corrective surgery have been presented with the goal of defining the modes of action for these intervention strategies. FE analysis has also been applied to simulate the effect of changes associated with the diabetic foot on factors such as blood supply to local tissues.

## Discussion

Existing models have not yet adequately captured the complexity of foot anatomy or the changes in tissue properties and structural factors associated with diabetes. Further work is required to improve the validity and credibility, of FE models and to standardize reporting. The development of patient-specific models is an extremely time-consuming process and reported run times are long. Progress in these areas is needed to ultimately move the technique beyond the research domain to allow it to become a clinically relevant tool at the patient level.

